# SmartFall: A Smartwatch-Based Fall Detection System Using Deep Learning

**DOI:** 10.3390/s18103363

**Published:** 2018-10-09

**Authors:** Taylor R. Mauldin, Marc E. Canby, Vangelis Metsis, Anne H. H. Ngu, Coralys Cubero Rivera

**Affiliations:** 1 Department of Computer Science, Texas State University, San Marcos, TX 78666, USA; trm119@txstate.edu (T.R.M.); vmetsis@txstate.edu (V.M.); 2 Department of Computer Science, Rice University, Houston, TX 77005, USA; marc.canby@gmail.com; 3 Department of Computer Science, University of Puerto Rico, San Juan 00927, Puerto Rico; coralys.cubero@upr.edu

**Keywords:** fall detection, deep learning, recurrent neural network, smart health, IoT application, IoT architecture, smartwatch

## Abstract

This paper presents SmartFall, an Android app that uses accelerometer data collected from a commodity-based smartwatch Internet of Things (IoT) device to detect falls. The smartwatch is paired with a smartphone that runs the SmartFall application, which performs the computation necessary for the prediction of falls in real time without incurring latency in communicating with a cloud server, while also preserving data privacy. We experimented with both traditional (Support Vector Machine and Naive Bayes) and non-traditional (Deep Learning) machine learning algorithms for the creation of fall detection models using three different fall datasets (Smartwatch, Notch, Farseeing). Our results show that a Deep Learning model for fall detection generally outperforms more traditional models across the three datasets. This is attributed to the Deep Learning model’s ability to automatically learn subtle features from the raw accelerometer data that are not available to Naive Bayes and Support Vector Machine, which are restricted to learning from a small set of extracted features manually specified. Furthermore, the Deep Learning model exhibits a better ability to generalize to new users when predicting falls, an important quality of any model that is to be successful in the real world. We also present a three-layer open IoT system architecture used in SmartFall, which can be easily adapted for the collection and analysis of other sensor data modalities (e.g., heart rate, skin temperature, walking patterns) that enables remote monitoring of a subject’s wellbeing.

## 1. Introduction

The Internet of Things (IoT) is a domain that represents the next most exciting technological revolution since the Internet. IoT will bring endless opportunities and impact every corner of our planet. In the healthcare domain, IoT promises to bring personalized health tracking and monitoring ever closer to the consumers. This phenomenon is evidenced in a recent Wall Street Journal article entitled “Staying Connected is Crucial to Staying Healthy” (WSJ, June 29, 2015). Modern smartphones and related devices now contain more sensors than ever before. Data from sensors can be collected more easily and more accurately. In 2014, it is estimated that 46 million people are using IoT-based health and fitness applications. Currently, the predominant IoT-based health applications are in sports and fitness. However, disease management or preventive care health applications are becoming more prevalent. The urgency for investment in health monitoring IoT technology is also echoed by a recent Wall Street Journal article (July 21, 2018) entitled “United States is Running Out of CareGivers”. By 2020, there will be 56 million people aged 65 and above as compared with 40 million in 2010. In [1], a system called VitalRadio is reported to be able to monitor health metrics such as breathing, heart rate, walking patterns, gait, and emotional state of a person from a distance. Recently, there is a surge in the number of the real-time preventive care applications such as those for detecting falls in elderly patients due to the aging population [2]. Previous work in fall detection required specialized hardware and software which is expensive to maintain. In [3], the authors reviewed 57 projects that used wearable devices to detect falls in elderly. However, only 7.1% of the projects reported testing their models in a real-world setting. The same paper also pointed out that a wearable wristwatch for fall detection has the added benefit of being non-intrusive and not incurring any additional injuries during a fall. Indeed, the main challenge for fall detection is the ability to create a highly accurate detection model that can run on unobtrusive and inexpensive devices. Sensors attached to the torso of the monitored subject have shown the ability to achieve higher detection accuracy; however, often in real life the elderly refuse to wear such sensors both for practical and psychological reasons.

We investigated both traditional (Support Vector Machine and Naive Bayes) and non-traditional (Deep Learning) machine learning techniques for the creation of fall models using three different datasets. Two of the datasets were collected by our team using a Microsoft Band 2 smartwatch [4], and a Notch [5] sensor. The dataset contains different simulated fall events and activities of daily living (ADLs) performed by a group of volunteer test subjects. The third dataset contains data coming from the Farseeing real-world fall repository [6]. The Smartwatch dataset is clearly needed since we are using a smartwatch device. Knowing the performance of our model on this dataset helps with the creation of a model that can be used in real life. The Farseeing dataset was the only one containing real falls from elderly people. While this dataset is not collected using smartwatch devices, it is still useful to compare how the Deep Learning model performs and compares against the traditional models. Evaluation of the models on Farseeing also helps to gain the insight on how the models deal with activity data that would realistically be seen with elderly people. The Notch dataset contains a much wider variety of ADLs collected by a wrist-mounted Notch sensor. Performance metrics for this dataset gives us a better idea of how the models perform and compare on a more complex wrist dataset. Notch also allows for a more precise labeling mechanism. Using the application provided to record Notch data, a user can visualize the data after it is recorded and set labels where falls happened at specific times. This helps examine the possibility that the model is being restricted by inaccurate labeling on the other two datasets. More information about each dataset is provided in Section 4.1.

In both the offline and online/real-time tests, Naive Bayes (NB) achieved a higher recall as compared with Support Vector Machine (SVM) under the traditional machine learning technique across the three datasets. Among the two traditional models, SVM is better in classifying ADLs, but it misses many critical falls. To the best of our knowledge, this is the first effort to conduct an in-depth study on using the Deep Learning (Deep) model, a non-traditional machine learning technique for fall detection using a wrist-worn watch. Our results show that a Deep Learning model for fall detection generally outperforms more traditional models across the three datasets.

As noted in the literature, a significant danger with falling in elderly adults is the inability to get up after the fall, which is reported to occur in 30% of the time. Currently, there are around eight million adults aged 65 and over that use medical alert systems like LifeLine, Medical Guardian and Life Alert [7]. The average cost of using such a system is 25 dollars per month. Our system is developed as an open source project and SmartFall will be offered as a free app. Another major problem in using these medical alert system is that, there is the danger that the person might not be conscious to press the Life Alert’s emergency button after a bad fall. With our SmartFall system, the detection of the fall in real time and the ability of sending a text message and a GPS location to a trusted family member, friend, or call 911 in real time ensure a better survival or improved care for the subject after a fall. The main contributions of the paper are:

- An in-depth study of both traditional and non-traditional machine learning algorithms for fall detection on three different fall datasets.
- A demonstration that the fall detection model trained using deep learning has better accuracy than models trained using either SVM or NB in predicting falls based on live wrist-worn acceleration data tested in both offline and online/real-time experiments.
- A three-layer open IoT system architecture and a real-time fall detection app that is privacy preserving and has an intuitive user interface (UI) for use by the elderly.

The remainder of this paper is organized as follows. In Section 2, we review the existing work on fall detection and emphasize on research works that specifically address fall detection using wearable devices. In Section 3, we provide a detailed description of the system architecture of our fall detection framework and the design of the UI. In Section 4, we describe the three fall datasets we used for fall detection and present our fall detection methods. In Section 5, we present the evaluation of the SVM, NB and Deep Learning models in both online and offline experiments, and finally in Section 6, we present our conclusion and future work.

## 2. Related Work

The World Health Organization (WHO) reported that 28–35% of people aged 65 and above fall each year. This rate increases to 32–42% for those over 70 years of age. Thus, a great deal of research has been conducted on fall detection and prevention. The early works in this area were concentrated on specially built hardware that a person could wear or installed in a specific facility. The fall detection devices in general try to detect a change in body orientation from upright to lying that occurs immediately after a large negative acceleration to signal a fall. Those early wearable devices are not well-accepted by elderly people because of their obtrusiveness and limited mobility. However, modern smartphones and related devices now contain more sensors than ever before. Data from those devices can be collected more easily and more accurately with the increase in the computing power of those devices. Smartphones are also widespread and widely used daily by people of all ages. There is thus a dramatic increase in the research on smartphone-based fall detection and prevention in the last few years. This is highlighted in the survey paper [8]. The smartphone-based fall detection solutions in general collect accelerometer, gyroscope and magnetometer data for fall detection. Among the collected sensor data, the accelerometer is the most widely used. The collected sensor data were analyzed using two broad types of algorithms. The first is the threshold-based algorithm which is less complex and requires less computation power. The second is the machine learning-based fall detection solutions. We will review both type of works below.

A threshold-based algorithm using a trunk-mounted bi-axial gyroscope sensor is described in [9]. Ten young healthy male subjects performed simulated falls and the bi-axial gyroscope signals were recorded during each simulated fall. Each subject performed three identical sets of 8 different falls. Eight elderly persons were also recruited to perform ADLs that could be mistaken for falls such as sitting down, standing up, walking, getting in and out of the car, lying down and standing up from bed. The paper showed that by setting three thresholds that relate to the resultant angular velocity, angular acceleration, and change in trunk angle signals, a 100% specificity was obtained. However, there was no discussion on the practicality of attaching a trunk-mounted sensor on a person for a prolonged period. The restriction on the mobility of people and the privacy issue of data storage were not discussed as well. There is also research work utilizing a thresholding technique set to only detect falls resulting in acceleration greater than 6 G (Gravity). While this will work very well for “hard” falls, we find that many of our falls were far below 6 G, producing around  3.5 G. A wrist-mounted device may encounter even smaller acceleration than 3.5 G if the subject does not use their hands to stop their fall. This type of fall is of special importance because an injury is more likely as the fall was not “caught” by the faller’s hands. This is one of the reasons machine learning approaches are considered more robust than thresholding techniques. Even though in controlled conditions thresholding techniques may appear to be superior, they often do not perform well on anomalous data, such as falls that only reach a maximum force of 3.5 G.

A promising use of machine learning algorithms is recently presented by Guirry in [10] for classifying ADLs with 93.45% accuracy using SVM and 94.6% accuracy using C4.5 decision trees. These ADLs include: running, walking, going up and down stairs, sitting and standing up. Their setup includes a Samsung Nexus Galaxy smartphone and the Motorola MOTOACTV smartwatch. Data were collected from the accelerometer, magnetometer, gyroscope, barometer, GPS, and light sensors. They synthesized a total of 21 features from all the sensors. They did not specifically address the fall detection problem.

The SVM learning algorithm has also been used for fall detection by other scholars in [11]. These scholars used a trunk-mounted tri-axial sensor (a specialized hardware) to collect data. They were able to achieve 99.14% accuracy with four features using only high-pass and low-pass accelerometer data. They used a 0.1 s sliding window to record minimum and maximum directional acceleration in that time period for a feature. We drew inspiration from this approach as it allowed us to access temporal data within each sampling point rather than having to choose a generalized feature for the whole duration which might not reflect a true fall. Other work in fall detection has focused on using multiple sensors attached to the subject. For instance, sensors can be placed on the lapel, trunk, ankle, pocket, and wrist. These systems typically show marvelous results of 100% accuracy but lack convenience, portability, and are more computationally intense for a smartphone due to more data being collected and processed.

In [12], a fall detection system architecture using multiple sensors with four traditional machine learning algorithms (SVM, NB, Decision Tree and KNN) was studied. The paper is the first to propose using ANOVA analysis to evaluate the statistical significant of differences observed by varying the number of sensors and the choice of a particular machine learning algorithm. The main conclusion from this paper is that sensors placed close to the gravity center of the human body (i.e., chest and waist) are the most effective. A similar paper in [13] conducted a study on the effect of the sensor location on the accuracy of fall detection. They experimented with six different traditional machine learning algorithms including dynamic time warping and artificial neural network. They showed that 99.96% sensitivity can be achieved with a waist sensor location using the KNN algorithm. Our work is focused on using a wrist-worn watch as the only sensor and thus cannot leverage these research results on other sensor locations.

A recent paper [14] on fall detection using on-wrist wearable accelerometer data concluded that threshold-based fall detection system is a promising direction because of the above 90% accuracy in fall detection with the added bonus of reduced computation cost. We disagree with this because the dynamic of fall cannot be captured in any rule-based or threshold-based system. The paper also pointed out the lack of real-world validation of majority of fall detection systems which we want to address in this paper.

There has also been some work on using Recurrent Neural Networks (RNNs) to detect falls; however, to our knowledge, no such work uses accelerometer data collected by a smartwatch to detect falls. In [15], the authors describe an RNN architecture in which accelerometer signal is fed into 2 Long Short-Term Memory (LSTM) layers, and the output of these layers is passed through 2 feed-forward neural networks. The second of these networks produces a probability that a fall has occurred. The model is trained and evaluated on the URFD dataset [16], which contains accelerometer data taken from a sensor placed on the pelvis, and produces a 95.71% accuracy. The authors also describe a method to obtain additional training data by performing random rotations on the acceleration signal; training a model with this data gives an accuracy of 98.57%.

The authors in [17] also propose an RNN to detect falls using accelerometer data. The core of their neural network architecture consists of a fully connected layer, which processes the raw data, followed by 2 LSTM layers, and ending with another fully connected layer. They also have some normalization and dropout layers in their architecture. The authors train and test their model with the SisFall dataset [18], which contains accelerometer data sampled at 200 Hz collected from a sensor attached to the belt buckle. In order to deal with a large imbalance in training data, of which ADLs form the vast majority, the authors define a weighted-cross entropy loss function, based on the frequency of each class in the dataset, that they use to train their model. In the end, their model attains a 97.16% accuracy on falls and a 94.14% accuracy on ADLs.

Our work differs primarily from these two papers in that we seek to develop a fall detection model that obtains accelerometer data from an off the shelf smartwatch rather than specialized equipment placed near the center of the body. This presents several challenges not addressed in these papers’ methodology. Because of its placement on the wrist, a smartwatch will naturally show more fluctuation in its measurements than a sensor placed on the pelvis or belt buckle. The scholars in [18] also use accelerometer data sampled at a 200 Hz frequency obtained by specialized equipment; this is a significantly higher than the frequency used by our smartwatch, which samples at 31.25 Hz. We also have the additional restriction that the model we develop should not consume so many computational resources that it cannot be run on a smartphone. Thus, while there has been some work done on deep learning for fall detection, we have additional constraints that make these works not directly relevant for our purposes.

In summary, many different machine learning algorithms such as the SVM, NB, KNN, Decision Trees, and Neural Networks have been applied to fall detection with some success. However, very few of those models have been tested in real time and on a wrist watch. Recently, an Android Wear-based commercial fall detection application called RightMinder [19] was released on Google Play. While the goal of RightMinder is very similar to ours, no technical details are available on the accuracy of the fall detection model and the management of the collected sensor data. We installed RightMinder and tried with 10 different simulated falls, it only detected 5 out of the 10 falls.

## 3. System Architecture

**Figure 1:**
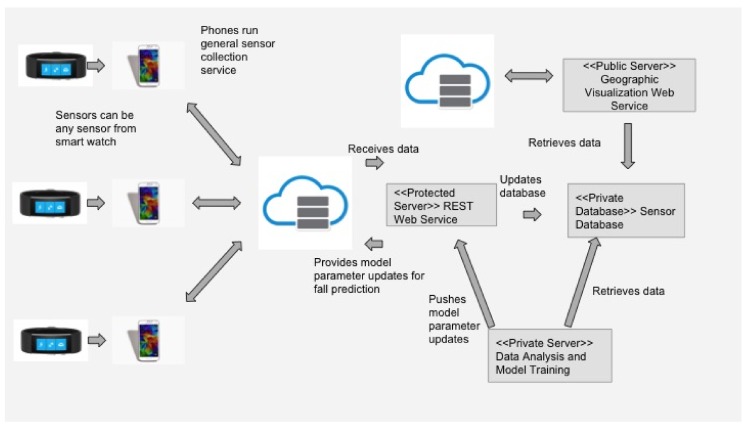
Architecture of SmartFall IoT system.

Figure 1 shows an overview of the IoT system architecture supporting our SmartFall application. It is a three-layered architecture with the smartwatch on the edge and the smartphone in the middle layer which runs the SmartFall application. In many IoT applications, it is critical that data can be stored locally to preserve privacy and is in close proximity to the program that processes and analyzes the data in real time. However, the smartphone has a limited storage and computation capacity and there is thus a need to periodically remove the sensed data or transfer the sensor data (with consent from user) to a server securely for continuous refinement of the fall detection model and for the long-term archival. The inner most layer serves as the heavy-duty computational platform which consists of multiple services including a web server to host applications that can visualize aggregated sensor data for public health education, a sensor database for archiving and visualizing sensed data from the smartwatch of the user who has given the consent (these data can be set up to be remotely accessible by the caregiver using a secure protocol that is HIPAA compliant), and machine learning services for analysis of the archived data for continuous refinement of the fall detection model. Data privacy is a big concern in health monitoring systems. In SmartFall, the data archived to the server are all de-identified and indexed by a randomly generated key which is only known to the watch wearer and the associated caregiver. It is impossible for any hacker to identify which sensor data is coming from which user on the server side.

This three-layer IoT system architecture is not specific to developing the fall detection IoT application. For example, we have also successfully used the same architecture for developing an IoT application for the prediction of blood alcohol content using the skin temperature and heart rate sensor data in [20]. In summary, this architecture has the potential to serve as a scalable service platform for IoT data for all kinds of devices and the associated applications.

Our long-term goal is to be able to support all existing smartwatches that can be paired with either Android or IOS-based smartphones. However, the Microsoft Band 2 was chosen as the wrist-worn device over other options in this prototyping phase due to the variety of sensors it supports and the low cost of acquiring the watch. The Microsoft Band 2 has the capability to track heart rate, galvanic skin response, barometric pressure, skin temperature, UV ray intensity, GPS location, skin capacitance, ambient light, sound, and, of course, it has an accelerometer and gyroscope. The Microsoft Band 2 also has multiple sampling rate options. The Nexus 5X smartphone was chosen to run our fall detection IoT application and receive sensor data from the smartwatch via a low-power Bluetooth communication protocol. This Nexus smartphone has a 1.8 GHz hexa-core processor and 2 gigabytes of RAM memory. This proved sufficient for real-time computation of the features, and for making the predictions, using models which were pre-trained offline.

### 3.1. Data Archiving and UI Design

We have implemented an archiving service which can be configured with a protocol where a participating user (with consent) can transmit all or selected sensed data in three minutes chunk to a designated server via a WiFi connection periodically. These sensor data can be used for the creation of other applications that provide other health monitoring benefit such as daily activities recognition and the overall wellbeing assessment of the user. Another critical service provided by the archiving service is the ability to transmit both false positive and true positive fall data via a REST-based web service periodically to a server. These archived false positive and true positive data samples can be used for re-training of the fall model and adapt the fall detection dynamically for a particular user.

**Figure 2:**
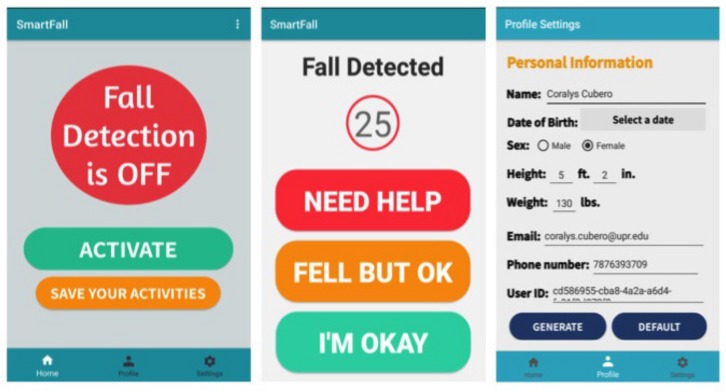
SmartFall User Interface.

When a fall has been detected by SmartFall on the phone, an alert text message can be sent to the carer upon confirmation by the user (via voice command on the phone or a simple interaction with the SmartFall as shown in Figure 2). The SmartFall interface is designed such that if a user is unconscious after a fall, the message can be configured to be sent automatically to the carer after a specified duration as shown in center screen in Figure 2. The alert message could include the user’s GPS location and other health metrics, such as the heart rate and the body temperature at the time of the fall. Figure 2 shows three of the UIs for the SmartFall application. The screen on the left shows the home screen UI for the application and the screen on the center shows the UI when a fall is detected. We followed the best practices advocated in [21] for the design of the UI for the elderly. The three main principles we adopted were strict color scheme with high contrasts, legible and big fonts, simple description of the system to engage them to use it.

We will briefly highlight some of the key features of SmartFall app. The home screen (left most screen in Figure 2) launches the SmartFall app when the user pressed the “ACTIVATE” button. A user can terminate the application by pressing the “SAVE YOUR ACTIVITIES” button. This will archive all the sensed data to the designated server and close the application. When a fall is detected, the screen in the middle of Figure 2 will be displayed with a programmable sound. The user is shown a screen with three buttons. The “NEED HELP” button will send a text message to the carer. The “FELL BUT OK” button will save the sensed data during that prediction interval as true positives. The “I’M OKAY” button will save these data as false positives. If the user did not interact with any of these three buttons, after a specified duration (e.g., 25 s), an alert message will be sent to the carer. The UI screen on the right most is for the one-time initialization of the user profile before the application can be launched. This UI includes setting up the contact details of the carer. Note that minimal personal data is collected and all those data are stored locally in the phone’s internal SQLite database. The automatically generated user-id is used by the system to differentiate different user’s data on the server. During data archiving, only this user-id and the selected sensed data such as the accelerometer data are sent to the server.

## 4. Methodology

### 4.1. Data Collection

It is impossible to collect simulated fall data from the elderly group of people which our application is targeted to because of higher likelihood of injuries. Currently, the only real-world fall data for elderly people (above 65 years old) available for research is from the Farseeing consortium [6]. This dataset contains a total of 23 falls sampled between 20 to 100 Hz using specialized sensors such as ActivePAL3 or McRobert Dynaport MiniMod. Since the sensor data of Farseeing was collected at various frequencies, we needed to resample all the data to 31.25 Hz, the frequency of our smartwatch. Resampling to the smartwatch frequency can help in answering the question of how well algorithms can perform at lower frequencies supported by low-power devices such as smartwatches. Performing tests on data with different frequencies is not as important to us, as it may not accurately reflect how our model would perform on real data received from a smartwatch at 31.25 Hz. We down-sampled the 100 Hz sensor data by removing random samples until the correct frequency of 31.25 Hz was obtained. The 20 Hz data was upsampled to 31.25 Hz by adding in random samples, where each sample was an average of the samples directly before and after it.

The Smartwatch dataset was collected from seven volunteers each wearing a MS Band watch. These seven subjects were all of good health and were recruited to perform simulated falls and ADLs. Their ages ranged from 21–55, height ranged from 5 ft to 6.5 ft. and the weight from 100 lbs to 230 lbs. Each subject was told to wear the smartwatch on his/her left hand and performed a pre-determined set of ADLs consisting of: jogging, sitting down, throwing an object, and waving their hands. This initial set of ADLs were chosen based on the fact there are common activities that involved movement of the arms. These datasets were automatically labeled as “NotFall”. We then asked the same subject to perform four types of falls onto a 12-inch-high mattress on the floor; front, back, left, and right falls. Each subject repeated each type of fall 10 times. We experimented with the sampling rates of 4 Hz, 1.25 Hz, and 62.5 Hz supported by the smartwatch, and settled with 31.25 Hz. We found that the data sampling frequency of 4 Hz, although it consumes fewer resources, missed too many critical sample points within the critical phase of a fall. On the other hand, the use of the higher sampling frequency of 62.5 Hz provided by the watch was flooding the application with too much data and incurred a high computation cost which is impractical for real-time prediction of falls. This dataset is available from http://www.cs.txstate.edu/~hn12/data/SmartFallDataSet under the smartwatch folder.

We implemented a data collection service on the smartphone to have a button that, when pressed, labels data as “Fall” and otherwise “NotFall”. Data was thus labeled in real time as it was collected, by the researcher holding the smartphone. However, the pressing of the button can introduce errors such as the button is being pressed too late, too early, or too long for a fall activity. To mitigate these errors, we post-processed the collected data to ensure that data points related to the critical phase of a fall were labeled as “Fall”. This is done by checking that for each fall data file, the highest peak of acceleration, and data points before and after that point, were always labeled as “Fall”.

The Notch dataset was collected from simulated fall data and ADLs using a wrist-worn Notch sensor [5]. The Notch system consists of multiple individual sensors which can be placed at different parts of the human body to collect motion data and reconstruct a full-body skeleton representation of the movements. We used the full array of sensors to reconstruct the movements for labeling purposes but utilized the data coming only from the sensor placed on the wrist for fall detection. We recruited seven volunteers with the age ranging from 20 to 35, heights from 5 ft to 6 ft and weights from 100 to 200 lbs. The Notch sensor is paired to an Android device (a tablet) via Bluetooth through a custom-built data collection app. After the Notch sensor is calibrated, it is given to a volunteer and strapped to his/her wrist. The sensor is calibrated once more while asking the volunteer to maintain a specific pose. Data collection is then initiated. A list of seven ADLs (sitting, getting up, jogging, throwing an object, waving, taking a drink, and going up and down stairs) and four type of falls (front, back, left, right) both soft and hard falls are read aloud for the volunteer to perform. When the list is finished the collection is stopped and the data is downloaded to the tablet from the sensors’ memory. This dataset is available from http://www.cs.txstate.edu/~hn12/data/SmartFallDataSet under the Notch folder.

### 4.2. Traditional Machine Learning Model

We experimented with two traditional machine learning algorithms. The first one is SVM, which is widely used in the literature for fall detection. The second one is NB, which is computationally more efficient. This efficiency in computation has also been observed by other authors in [22]. They demonstrated that NB could classify fall in less than 0.3 s as compared to Decision Tree which took more than 6 s.

#### 4.2.1. Feature Selection

For traditional machine learning algorithms (SVM and NB), before any learning can occur, the raw fall data collected must be processed to extract a set of features to be used for creating the model.

**Figure 3:**
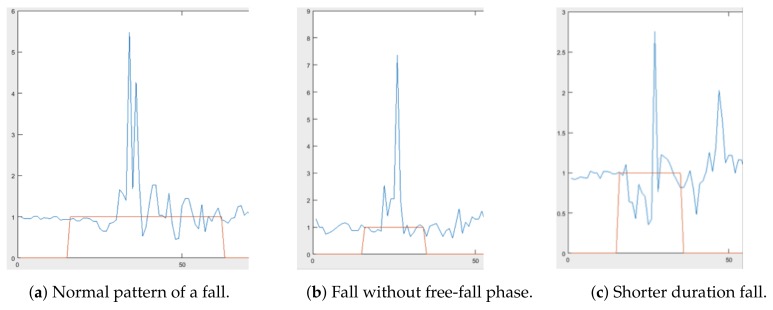
Different fall signal patterns.

In our experiments, four features we extracted, which are: (1) length of the acceleration vector at the time of sampling ($A_{\mathrm{res}}$), (2) minimum resultant acceleration in a 750 ms sliding window ($S_{\min}$), (3) maximum resultant acceleration in the same 750 ms sliding window ($S_{\max}$), and (4) the Euclidean norm of the difference between maximum and minimum acceleration in the same sliding window (ΔS). The determination of the four features that we used for training a model to recognize a fall was influenced by the concept of a critical phase of a fall, described in [23]. The critical phase of a fall encompasses the free-fall stage, the impact, and the dampening oscillations to rest. Figure 3a shows what we would expect to see from the definition of a normal fall which is defined by the critical phase. Note the height of the graph; the highest acceleration recorded for this fall was a fairly reasonable  5.5 G. We can also see the dampening oscillations in the latter half of the fall. However, because our accelerometer is wrist-mounted, not all falls follow this pattern. Figure 3b shows a fall that has a reasonable maximum acceleration (the peak), but almost completely lacks the weightlessness portion we would expect. In Figure 3c, the overall fall pattern is similar but the scale is completely different. The maximum acceleration never exceeded 3 G. These latter two examples may appear to be anomalies but are actually extremely prevalent especially for the left and right fall data we collected.

We used the Euclidean norm to measure the length (magnitude) of acceleration and velocity vectors. That is, for any vector $\vec{r}$ in $R^{3}$, we used:

(1) $${∥\vec{r}∥}_{2}=\sqrt{r_{x}^{2}+r_{y}^{2}+r_{z}^{2}}$$

$A_{\mathrm{res}}$, resultant acceleration, is defined as the magnitude of the acceleration vector at the start of a fall. Using Equation (1), we defined:

(2) $$A_{\mathrm{res}}={∥A∥}_{2}$$

ΔS, adapted from Liu and Cheng’s paper in [11], is the magnitude of the difference between minimum and maximum acceleration in a 750 ms sliding window. That is, using Equation (1), we defined:

(3) $$\Delta S={∥S_{\max}-S_{\min}∥}_{2}$$

where $S_{\min}$ and $S_{\max}$ adapted from Jantaraprim et. al’s paper [23] are defined as the minimum and maximum resultant acceleration in a sliding window of 750 ms. In the original implementation by Jantaraprim et al. and Liu and Cheng’s, the sliding window was designated to be 0.1 s. In the literature there is little consensus on what sliding window size is optimal for the calculation of $S_{\min}$ and $S_{\max}$. 0.2 to 2 s windows have been used but windows between about 0.5 and 1 s were more common. When computing over streaming data, the computation must accommodate data at the boundary of a sliding window and thus it is important to set an overlapping threshold. We experimented with various window sizes of 500 ms to 1000 ms and different overlaps from 90% to 50%. The combination of 750 ms window size with 50% overlap achieved the best accuracy rate.

SVM and NB models are trained to predict fall on a sample by sample basis, categorizing each sample as a fall or not a fall. This method does not necessarily suit the nature of the activities we are trying to detect as detecting a fall constitutes finding a pattern from a succession of sample points as shown in Figure 3. This means the final prediction of whether a movement of the wrist is a fall or not a fall must be derived by a second step which counts a range of consecutive positive fall labels within a prediction interval. To determine this range, we experimented with the model in real life using activities that could be defined in two categories: (1) short-term spikes in acceleration and (2) long-term increases in acceleration. Activities that could be categorized as (1) are various hand and arm gestures such as waving, throwing an object, and punching. An activity that would belong in category (2) would be running or exercising involving arm movements which is demarcated by a sudden increase in acceleration that is maintained over a duration of at least three seconds (i.e., longer than a typical fall). Our initial experimental result which is discussed in our earlier paper [24] shows a threshold between 3 and 50 as the ideal in the sense that it gives the highest recall and accuracy in fall prediction when tested. The pseudo codes of our traditional machine learning fall prediction depicted in Algorithm 1 consists of fall features computation, classification of each fall instance (using either SVM or NB), and the final prediction of fall or not fall by counting the number of consecutive fall predicted within a sliding window.
**Algorithm 1** Prediction algorithmInput: AccelerometerData ($A_{x},A_{y},A_{z}$) in CSV fileOutput: prediction of true or false of a fallslidingWindow=750 msconsecutiveCount=0prediction=false**for all**AccelerometerData in slidingWindow **do**  Compute $A_{\mathrm{res}}$, ΔS, $S_{\min}$, $S_{\max}$  Write to CSV file**end for**Initialize the prediction interval (i.e., which data sample in the CVS file to start the prediction)traditionModel = the trained NB or SVM fall detection model**for all**instance in predictionInteval **do**  label = traditionalModel.classifyInstance(instance)  **if** (label==″Fall″) **then**    ++consecutiveCount  **else if** (3<=consecutiveCount<=50) **then**    prediction=true
    consecutiveCount=0
  **else**
    consecutiveCount=0
  **end if**
**end for****return**prediction

### 4.3. Deep Learning Model

Next, we experiment with a non-traditional machine learning approach, namely Deep Learning, in order to evaluate its suitability for the task of fall detection and the possibility of achieving higher accuracy compared to the traditional algorithms.

One of the disadvantages of traditional machine learning algorithms is the need for a priori feature extraction from the data. Feature extraction and selection are tasks that need to be performed before any learning can occur. The types of features to be extracted have to be manually specified and thus their effectiveness heavily depends on the ingenuity of the researcher. Each signal has different temporal and frequency domain characteristics [25]. This makes feature extraction and selection a complicated task, which can heavily affect the performance of the machine learning model. For example, in [26] the accuracy of the SVM algorithm varies depending on the feature selection method used. In feature-dependent methods, the main difficulty is to extract the appropriate features. In certain types of data, to extract high quality features we need human-like understanding of the raw data.

Deep learning comes to solve this problem by eliminating the need for separate feature extraction, selection and model training phases. Deep learning refers to the process of machine learning using deep neural networks. Deep neural networks are biologically inspired variants of the Multiple Layer Perceptrons (MLPs) [27]. Deep learning has shown significant improvements in areas such as image classification and object detection. In early object detection approaches, people extracted features and fed these features to learning algorithms (e.g., SVM) to successfully detect objects of interest (e.g., pedestrians) in the image. However, when these methods were used to detect several classes other than pedestrians e.g., car, sign or tracks, the accuracy of the model dropped [28].

The use of deep convolutional neural networks (CNNs) showed a notable increase in the performance of detecting objects using highly challenging datasets [29]. The two most common implementations of deep neural networks are CNNs [29] and RNNs [30]. CNN is a type of feed-forward artificial neural network which takes fixed size inputs and generates fixed-size outputs. CNNs are ideal for images and video processing. RNNs, unlike feed-forward neural networks, can use their internal memory to process arbitrary sequences of inputs. RNNs use time-series information, which means that past data points can influence the decision regarding current data points. RNNs are ideal for the analysis of temporal data, such as speech and sensor data streams. In summary, due the sequential nature of the data points collected from accelerometers, RNNs are better suited to our fall detection task.

A popular variant of the traditional RNN contains units called gated recurrent units (GRU). A GRU network is similar to a LSTM network [31]. Both contain gating mechanisms that look to solve the vanishing gradient problem. GRU networks have been shown to converge as well as LSTM networks and require less computation as they do not contain as many trainable parameters. This is ideal for the computation constrained smartphone. This type of RNN network architecture underlies our Deep Learning model. It helps capture activities over a longer period of time so that they can be better distinguished from others. We believe it has an advantage over threshold-based algorithms for this reason. Many regular activities can briefly trigger high acceleration values, and threshold-based models often have a hard time telling these apart from falls. Looking at many data points at once allows us to make more robust distinctions between activities.

**Figure 4:**
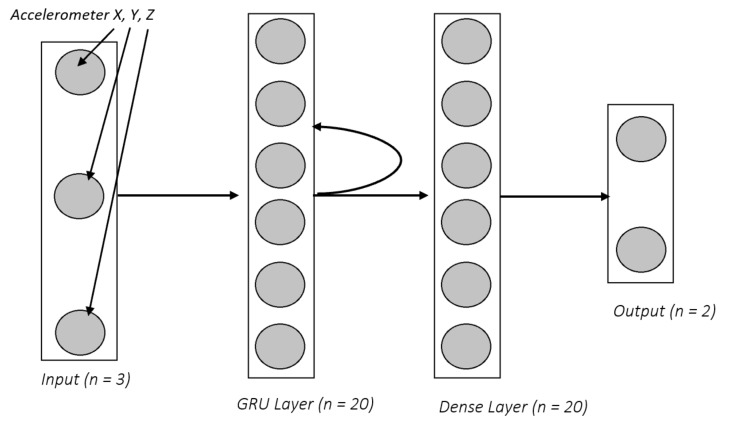
RNN Model Architecture.

Figure 4 displays our model architecture:

The model contains an input layer, two hidden layers, and an output layer. The input layer contains 3 nodes for the raw data; the accelerometer x,y,z vectors. It then feeds through our hidden layers: a recurrent layer (GRU) of size 20 ReLU nodes, and a fully connected layer of size 20 ReLU nodes. The output is a 2 node SoftMax layer which outputs a predicted probability that a fall has occurred. This model is lightweight relative to many deep learning architectures, and makes inference computation much more efficient for mobile devices. RNNs are traditionally trained with backpropagation through time (BPTT), so it is necessary to specify how many steps *n* in the past the network should be trained on. This parameter is important since our falls and activities occur over a period of time. If we train the network on only a few steps in the past, it will not capture the full scope of the activity. However, if we train it over too many steps, the network may take into account past accelerometer data that is not relevant. We settled on using n≈40 steps for this model. This means each prediction the model makes takes into account ≈1.28 s of data. This is enough time to capture the aspects of a fall, as well as to rule out some activities as falls.

**Figure 5:**
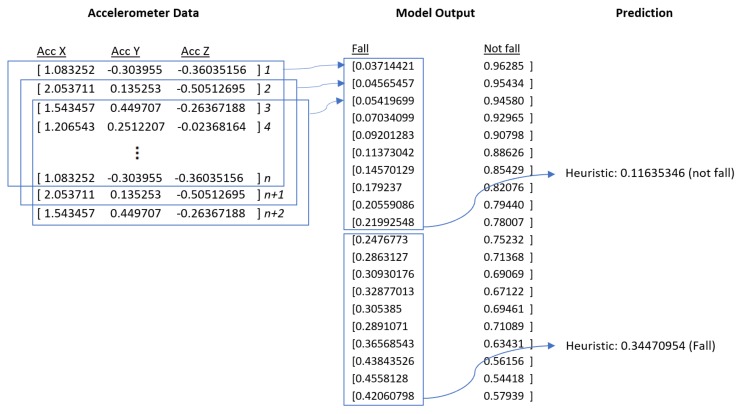
Prediction scheme for deep learning.

Model predictions (i.e., predictions produced by the neural architecture) begin once the number of sensor data points acquired is equal to the number of steps. Every model prediction thereafter will only require one additional data point, as the model will slide one data point at a time, reusing all of the previous data points except for the least recent. However, before producing a final prediction, we generate a heuristic value based on the probabilities produced by several model predictions. We compute the average value of 10 consecutive probabilities, and compare this with a pre-defined threshold value. If the average probability breaches this threshold, then it is considered a fall prediction. This helps to avoid isolated positive model predictions from triggering a false positive. Figure 5 outlines this schematic.

## 5. Evaluation

Our goal is to be able to detect accurately whether someone has fallen in real time based on the motion sensed by a smartwatch that a person is wearing on their wrist. We do not want to miss a fall, which implies a fall detection model with a high Recall or Sensitivity. A missed fall is represented in our evaluation experiments as a false negative (FN). We also do not want to have too many false alarms, which in our evaluation as represented as false positives (FPs), and thus, we want to achieve a high precision. In particular recall, precision, and overall accuracy are calculated as:

Recall=Sensitivity=TP/(TP+FN)

Precision=TP/(TP+FP)

Accuracy=(TP+TN)/(TP+TN+FP+FN)

where true positives (TP) is the number of correctly detected falls. The number of True Negatives (TN) is not of particular interest to this application as negative instances represent non-falls and, in practice, they greatly outnumber the number of positive instances.

In this section, we first present our method for evaluating a model on the three datasets described in Section 4. We then present the results of training and evaluating three models—NB, SVM, and Deep Learning—on the datasets. We also discuss the results of running these three models in real time with volunteers wearing smartwatches. We conclude with a comparison of the three models, and what this means for our Deep Learning model.

The three datasets on which we train and evaluate the three models are the Farseeing, Smartwatch, and Notch datasets, described in Section 4. Each dataset contains continuous accelerometer data, and each data point is marked with “Fall” or “NotFall”. For our purposes, a “Fall” instance is a series of consecutive data points marked “Fall”—this corresponds to the interval of time in which a person is falling. The Smartwatch dataset is also labeled with ADL information, which makes it easy to tell where each “ADL” instance starts and stops. The Notch and Farseeing datasets, however, are not labeled with ADL intervals; they have only continuous accelerometer data marked with “NotFall”. Thus, it is harder to determine intervals in which a single ADL occurs, since ADLs typically appear back-to-back. Therefore, for these datasets, we will consider an “ADL” instance to be a 1-s interval of consecutive data points marked “NotFall”. Because there are many more data points marked “NotFall” than “Fall” in the Notch and Farseeing datasets, this formulation will produce far more ADL activities than falls. The number of falls and ADLs in each dataset is given in Table 1.

As described in Section 4.2.1, a NB or SVM model makes a final fall prediction when the algorithm outputs between 3 and 50 consecutive data points predicted as fall. We determine if this prediction is correct by checking if any of the predicted consecutive falls match the label on the corresponding row of the dataset. We do a similar process to determine if a Deep Learning model’s fall prediction is correct. As described in Section 4.3, a Deep Learning model produces a prediction after computing the heuristic, which is the average of probabilities generated by the neural network architecture over 10 windows of *n* steps, after comparing this heuristic to a pre-defined threshold. When a Deep Learning model makes a fall prediction, we determine if that prediction is correct by checking the labels in the dataset corresponding to the final row of each of the 10 windows. If any of these labels is a “Fall”, the prediction is deemed to be correct.

The results of training and evaluating a NB, SVM, and Deep Learning model on the three datasets are presented in Table 2. For the Smartwatch dataset, each model is trained on two-thirds of the data and is tested on the remaining third. The Notch and Farseeing datasets are analyzed using a leave-one-out strategy where the models are trained on all user files but one. Even though the data in these datasets is pre-recorded, we simulate an online environment by processing the data as if it were being received live from a smartwatch. This effectively allows us to test various models in a real-world situation without the expense of constantly recruiting volunteers.

The NB model demonstrates a recall greater than 0.8 on all three datasets—this demonstrates that the model does well predicting falls. However, the model has a considerably lower precision on each dataset, indicating a lot of FPs. In particular, the precision on the Farseeing dataset is 0.005; this especially low number is a result of poor performance in the face of imbalanced data. In the Farseeing dataset, there are over 1000 ADLs for each fall; in the Notch dataset, which is also imbalanced, there are only 23 ADLs for each fall—this increase in imbalance is enough to substantially lower the precision value between the two datasets despite only slightly lower accuracies on falls and ADLs. It is also worth noting that the imbalance in fall and ADL data in the Notch and Farseeing datasets causes the overall accuracy to be dominated by the ADL accuracy on all three models.

The SVM model demonstrates a similar pattern to the NB model in that it generally has a high recall and a lower precision. With the exception of the Farseeing dataset, the SVM model has a recall greater than or equal to 0.8, indicating that it does pretty well on falls. The low recall of 0.55 on the Farseeing dataset challenges this; this may suggest that the model has a hard time identifying a rare class in the midst of extremely imbalanced data. Like the NB model, the SVM also demonstrates a lower precision value on every dataset, suggesting there are a lot of FPs. We believe that the primary cause of this in both the NB and SVM models is that these models use derived acceleration features rather than raw accelerometer data. This fundamentally limits the models, as they cannot discern between certain directional characteristics of ADLs and falls that may only be accessible through raw accelerometer data.

The Deep Learning model outperforms the NB and SVM model on every metric except for precision on the Farseeing dataset, which is lower than the SVM precision. On the Smartwatch and Farseeing dataset, the deep model has perfect recall, and it has a 0.89 recall on the Notch dataset. This demonstrates that it does a very good job in identifying falls. Like the NB and SVM models, the precision values for the deep model are comparatively lower than the corresponding recall values, suggesting that it also struggles with FPs. However, with the exception of a slightly lower precision than the SVM on the Farseeing data, the deep model’s precision is higher than both NB and SVM; we believe that this is because the deep model is trained on the raw accelerometer data, which allows it to extract helpful signals of ADLs that are not present in the derived acceleration features given to the NB and SVM models. Also, like the NB model but unlike the SVM model, the deep model has a substantially lower precision on the Farseeing dataset than the other two datasets; we believe that the high level of imbalance in the Farseeing dataset may be behind this anomaly, especially as both the fall accuracy and the ADL accuracy are considerably greater on the Farseeing dataset than either the Smartwatch or Notch datasets.

In addition, we evaluated the same three models in real time by recruiting five volunteers of various heights and weights who recorded each model’s predictions on various falls and ADLs. In this case, each model was trained on the entire Smartwatch dataset described in Section 4 and tested on the volunteers, who each wore a smartwatch paired with the smartphone app. Each volunteer placed the smartwatch on his or her left wrist, and was asked to do five each of front, back, left, and right falls. To see how the model performs on non-fall data, each volunteer also performed five each of sitting, waving (3 s), jogging (10+ s), and walking (10+ s) ADLs. Testing in this way lets us evaluate the model’s capabilities in a true online situation as well as seeing how each model performs on specific kinds of falls and ADLs. The other datasets are only labeled as “Fall” or “NotFall”, so it is not possible to analyze which types of falls and ADLs the model detects. The real-time results can be found in Table 3.

Our NB model detects falls at a reasonable rate. Its detection rate, however, varies across the different types of falls. Front and left falls are both detected by the NB model at a rate above 80%. Back fall accuracy drops to 60%, and right falls are the lowest at 40%. The fact that the model performs much better on left falls than right falls suggests that the model may be sensitive to the wrist the smartwatch is placed on. Furthermore, the model’s poorer performance on back falls suggests that it performs like a threshold-based algorithm, since the wrist movement in back falls is not as intense. Another threshold-like behavior for this model is the tendency to perform well on light ADLs like sitting and walking (obtaining over 93% accuracy on these activities), but poorly on more motion-intensive ADLs like waving and jogging (obtaining less than 50% accuracy on these).

Our SVM model obtained much different results than the other two models. It scores the best on nearly every ADL category, while performing quite poorly on falls themselves. We prioritize obtaining TPs over not obtaining FPs, and so we consider our SVM model to have the worst performance by a wide margin. Like the NB model, the SVM model behaves like a threshold-based algorithm; however, it is far less sensitive than NB. This can be seen when looking at the ADL accuracies. While the SVM performs the best of the three models on jogging and waving, it still performs worse on these activities than it does on sitting and walking. The biggest difference between SVM and NB is the fall results: the SVM performed the same on right falls and left falls, while NB performed better on left falls. This suggests that our SVM model adapts better to different wrist placement, despite its overall poor performance.

Our Deep Learning model performs the best on detecting falls. It is far better than the other models at detecting falls with more unique wrist movements, such as back falls. Whereas both the SVM and NB do quite poorly on back falls, the Deep Learning model detected back falls as well as the other fall types. However, the Deep Learning model is not very accurate on ADLs. In ADLs that contained quick and abrupt motion, such as jogging and waving, the Deep Learning model performed slightly worse than the SVM. Unlike the SVM and NB models, however, the Deep Learning model struggles more with lighter activities such as sitting and walking. For this reason, the Deep Learning model can quickly be separated from threshold-based algorithms: it distinguishes well between quick intense movements (falling) and intense movements over a period of time (jogging/waving), but does not distinguish lighter movements (sitting/walking) from the intense movements well. Along with the good fall detection results, this is a strong indicator that the Deep Learning model does not rely on just high acceleration values for its predictions. Feature extraction is a likely contributor to this limitation for the NB and SVM models. The features that these models are trained with are all a direct function of the resultant acceleration. We believe that the use of raw accelerometer data for our Deep Learning model creates a much wider range of correlations that the model can pick up on. These additional correlations can help the model avoid regressing on a single feature, such as just a high acceleration value.

The Deep Learning, SVM, and NB models performed better overall on the offline Smartwatch dataset than on the real-time experiments. One possible explanation for this is that many of the participants in the Smartwatch dataset were included in both the training and testing of the models, making it easier for the models to recognize patterns in the falls. The real-time experiments, however, were tested on volunteers, whose data was not used to train the model. Thus, it is natural that the models do not perform as well in the online setting.

It is also important to notice that the Deep Learning model generalizes much better to new volunteers than the NB and SVM models. The SVM detected 85.7% of falls on the Smartwatch dataset, dropping to 26% in real time, while NB detected 92.3% of falls on the Smartwatch data, dropping to 66% in real time; the Deep Learning model, however, dropped from a 100% detection rate to an 86% rate. A potential cause for this is feature extraction. Both models that use extracted features showed a significant performance drop in falls detected. This may indicate the extracted features are removing important patterns in the raw data, thus oversimplifying the prediction and causing it to generalize poorly. The Deep Learning model, which uses raw data, has the opportunity to learn patterns that may help it to generalize.

In summary, our results show that a Deep Learning model for fall detection generally outperforms more traditional models such as NB and SVM. Offline and online results indicate that our deep model has both higher precision and higher recall than the NB and SVM models. We believe that this is due to the deep model’s ability to learn subtle features from the raw accelerometer data that are not available to NB and SVM, which are restricted to learning from a small set of extracted features. Furthermore, the deep model exhibits a better ability to generalize to new users when predicting falls, an important quality of any model that is to be successful in the real world. Despite the overall success of the Deep Learning model, it still has challenges, primarily in its failure to properly classify ADLs, particularly lighter ones such as sitting and walking. We believe that with further adjustments to the deep learning architecture and parameters, it will be able to detect these motions at least as well as its NB and SVM counterparts.

## 6. Conclusions

Many fall detection systems have been experimented and implemented in the last few years using specialized sensors which are expensive and inconvenient to use. We believe the simplicity of only having to wear a smartwatch would not only reduce costs, but would also be very convenient and non-intrusive for the users. We performed in-depth evaluation of both traditional and non-traditional machine learning algorithms using three datasets in order to determine what model will work best on a wrist-mounted smartwatch’s accelerometer data for fall detection. The traditional machine learning algorithms require pre-processed accelerometer data based on four selected features which are $A_{\mathrm{res}}$, ΔS, $S_{\max}$ and $S_{\min}$. This pre-processing of the data resulted in the loss of important contextual information. As discussed in Section 5, both NB and SVM do not generalize well as demonstrated with the online/real-time test results of a recall of 26% for SVM and 66% with NB. Deep Learning can use the raw accelerometer data without any pre-processing and achieve almost 100% accuracy using the Smartwatch and the Farseeing datasets in the offline tests and only dropped to 86% with the online test. In particular, the deep recurrent neural network works well on time-series data by considering past data points when making a single prediction. Deep Learning is also able to learn subtle features from the raw accelerometer data that are not available to NB and SVM and is the most accurate in fall detection using a wrist-worn watch. Our study also demonstrated that SVM while being a popular model cited in various literatures for fall detection has the worst true positive rate of fall detection across the three datasets. The 86% accuracy for deep learning is the best we have obtained based on our real-world test. However, the medical field is often averse to a device that only works “most of the time”. Therefore, it is our goal to further improve both the recall and precision of the Deep model by making further adjustments to the deep learning architecture and parameters. We also plan to archive both true positive and false positive data collected in our planned comprehensive real-world test to continuously re-train the model.

In addition, we developed a real-time fall detection app called SmartFall running on an Android phone with an intuitive UI for elderly people. SmartFall allows us to perform real-time/online test of the three (NB, SVM, Deep) models from smartwatch’s accelerometer data with ease. Our current real-time test just consists of asking new volunteers who did not contribute to the original smartwatch dataset to perform scripted falls and ADLs using SmartFall. While this is by no mean a real-world test, we gained important insight on the performance of the three models on different type of falls and type of ADLs from this test. One of our immediate future works is to conduct a comprehensive real-world test using SmartFall in a nursing home. In particular, we aim to use SmartFall to collect ADLs data from the elderly and verify how many of those ADLs are falsely classified as falls to judge the practically of our application. We will recruit five seniors for the test. Some of the questions we intent to study are: (1) How long does each prediction take in real time? (2) How long will the battery last on the smartwatch and the smartphone by running SmartFall continuously together with all the other apps used regularly in a day by a senior? (3) How many hours seniors will typically wear smartwatches in a day? (4) How much ADL data can be collected in a day from one senior? (5) Which ADL activity generates the most FPs? (6) What are seniors’ main concerns regarding wearing smartwatches and carrying smartphones around for fall detection?

SmartFall is designed to use an open three layers architecture which can be generalized to a plug and play service platform for the development of other health IoT applications. For example, our real-time collection of accelerometer data and the data labeling process can be reused for the development of an IoT application that needs to track the arm movement of ADHD (Attention Deficit Hyperactivity Disorder) children and provide early diagnosis and alerts.

We acknowledge that our fall detection model is trained using data from healthy volunteers, which might not reflect the actual fall data from elderly people. It is impossible to collect simulated fall data from the elderly group of people because of higher likelihood of injuries. We overcome that by performing the evaluation of our models with the Farseeing dataset which contain real falls from elderly people. The result in Table 2 shows that the model performance is not dependent on specific accelerometer data.

## Figures and Tables

**Table 1:** Number of falls and ADL’s by dataset.

	# Falls	# ADLs
Smartwatch	91	90
Notch	107	2456
Farseeing	23	27,412

**Table 2:** Offline results by algorithm on 3 datasets.

		NB	SVM	Deep
Smartwatch	Precision	0.60	0.68	0.77
	Recall	0.92	0.86	1.0
	ADL Acc.	0.38	0.60	0.70
	Overall Acc.	0.65	0.73	0.85
Notch	Precision	0.58	0.53	0.79
	Recall	0.89	0.80	0.89
	ADL Acc.	0.97	0.97	0.99
	Overall Acc.	0.97	0.96	0.99
Farseeing	Precision	0.005	0.44	0.37
	Recall	0.82	0.55	1.0
	ADL Acc.	0.87	0.99	0.99
	Overall Acc.	0.87	0.99	0.99

**Table 3:** Online results by algorithm.

		NB	SVM	Deep
Falls	Back	0.60	0.20	0.84
	Front	0.84	0.28	0.80
	Left	0.80	0.28	0.96
	Right	0.40	0.28	0.84
ADLs	Sitting	0.93	0.97	0.40
	Waving	0.40	0.76	0.60
	Jogging	0.8	0.72	0.64
	Walking	1.00	0.92	0.56
Overall	Precision	0.62	0.62	0.64
	Recall	0.66	0.26	0.86
	Accuracy	0.64	0.56	0.70
